# Alcohol use among adolescents in India: a systematic review

**DOI:** 10.1017/gmh.2021.48

**Published:** 2022-01-07

**Authors:** Abhijit Nadkarni, Allison Tu, Ankur Garg, Devika Gupta, Sonal Gupta, Urvita Bhatia, Niharika Tiwari, Anna Heath, Karen Wen, Godwin Fernandes, Richard Velleman

**Affiliations:** 1 Centre for Global Mental Health, London School of Hygiene and Tropical Medicine, Keppel Street, London, UK; 2 Addictions Research Group, Sangath, Porvorim, Goa, India; 3 Department of Psychology, Harvard University, 33 Kirkland Street, Cambridge, USA; 4 Oxford Brookes University, Oxford, UK; 5 Tees, Esk and Wear Valleys NHS Foundation Trust, Darlington, UK; 6 Social Finance, Tintagel House, 92 Albert Embankment, London, UK; 7 Department of Psychology, University of Bath, Bath, UK

**Keywords:** Adolescents, alcohol, alcohol use disorders, India, systematic review

## Abstract

**Background:**

Alcohol use is typically established during adolescence and initiation of use at a young age poses risks for short- and long-term health and social outcomes. However, there is limited understanding of the onset, progression and impact of alcohol use among adolescents in India. The aim of this review is to synthesise the evidence about prevalence, patterns and correlates of alcohol use and alcohol use disorders in adolescents from India.

**Methods:**

Systematic review was conducted using relevant online databases, grey literature and unpublished data/outcomes from subject experts. Inclusion and exclusion criteria were developed and applied to screening rounds. Titles and abstracts were screened by two independent reviewers for eligibility, and then full texts were assessed for inclusion. Narrative synthesis of the eligible studies was conducted.

**Results:**

Fifty-five peer-reviewed papers and one report were eligible for inclusion in this review. Prevalence of ever or lifetime alcohol consumption ranged from 3.9% to 69.8%; and prevalence of alcohol consumption at least once in the past year ranged from 10.6% to 32.9%. The mean age for initiation of drinking ranged from 14.4 to 18.3 years. Some correlates associated with alcohol consumption included being male, older age, academic difficulties, parental use of alcohol or tobacco, non-contact sexual abuse and perpetuation of violence.

**Conclusion:**

The evidence base for alcohol use among adolescents in India needs a deeper exploration. Despite gaps in the evidence base, this synthesis provides a reasonable understanding of alcohol use among adolescents in India and can provide direction to policymakers.

## Introduction

According to the Global Burden of Diseases, Injuries, and Risk Factors Study (GBD) 2019, among adolescents and young adults (aged 10–24 years), alcohol-attributable burden is second highest among all risk factors contributing to disability-adjusted life years in this age group (GBD 2019 Risk Factors Collaborators, 2020). The exposure of the adolescent brain to alcohol is shown to result in various cognitive and functional deficits related to verbal learning, attention, and visuospatial and memory tasks, and behavioural inefficiencies such as disinhibition and elevated risk-taking (Spear, 2018). Alcohol consumption in adolescents results in a range of adverse outcomes across several domains and includes road traffic accidents and other non-intentional injuries, violence, mental health problems, intentional self-harm and suicide, HIV and other infectious diseases, poor school performance and drop-out, and poor employment opportunities (Hall *et al*., 2016).

Adolescence is a critical period in which exposure to adversities such as poverty, family conflict and negative life experiences (e.g. violence) can have long-term emotional and socio-economic consequences for adolescents, their families and communities (Knapp *et al*., 1999; Knapp *et al*., 2002). Substance use, including alcohol, is typically established during adolescence and this period is peak risk for onset and intensification of substance use behaviours that pose risks for short- and long-term health (Anthony and Petronis, 1995; DeWit *et al*., 2000; Hallfors *et al*., 2005; Schmid *et al*., 2007; Hadland and Harris, 2014). As such, early initiation of alcohol use among adolescents can provide a useful indication of the potential future burden among adults including increased risk for academic failure, mental health problems, antisocial behaviour, physical illness, risky sexual behaviours, sexually transmitted diseases, early-onset dementia and the development of alcohol use disorders (AUDs) (Hingson *et al*., 2006; King and Chassin, 2007; Dawson *et al*., 2008; Nordström *et al*., 2013).

India continues to develop rapidly, and accounts for most of the increase in alcohol consumption per capita for WHO's South-East Asia region (World Health Organization, 2018). Although India has a relatively high abstinence rate, many people who do drink are either risky drinkers or have AUDs (Benegal, 2005; Rehm *et al*., 2009). Finally, the existing policies in India have failed to reduce the harm from alcohol because the implementation of alcohol control efforts is fragmented, lacks consensus, is influenced by political considerations, and is driven by narrow economic and not health concerns (Gururaj *et al*., 2021).

India has the largest population of adolescents globally (253 million people aged 10–19 years), constituting 21% of the population (Government of India, 2011; Boumphrey, 2012). Additionally, adolescents as young as 13–15 years of age have started consuming alcohol in India (Gururaj *et al*., 2016). Despite this growing public health problem, the official policy response in India remains primarily focused on AUDs, particularly alcohol dependence in adults, with an absolute disregard for the potential of prevention programmes. One potential reason for this is the limited understanding of the onset and progression of alcohol use and AUDs amongst adolescents in India. The aim of this paper is to bridge that knowledge gap by synthesising the evidence about the prevalence and correlates of alcohol use and AUDs in adolescents from India.

The specific objectives are to examine the following in adolescents from India: (a) prevalence of current and lifetime use of alcohol, (b) prevalence of current AUDs, (c) patterns (e.g. frequency, quantity) of alcohol use, (d) sociodemographic, social and clinical correlates of alcohol use and AUDs, and (e) explanatory models of and attitudes towards alcohol use and AUDs, e.g. perceptions of the problem and its causes. This paper synthesises the evidence about alcohol and AUDs using data from a comprehensive review that we conducted of any substance use and substance use disorders amongst adolescents in India.

## Methods

### Design

*Systematic review*. The review protocol was registered prospectively on Prospero (registration ID CRD 42017080344).

### Inclusion and exclusion criteria

There were no limits placed on the year of publication of the paper, gender of the participants and study settings in India. We only included English language publications as academic literature from India is predominantly published in such publications. Adolescents were defined as anyone between 10 and 24 years of age (Sawyer *et al*., 2018). Studies reporting alcohol use and/or AUDs in a wider age range (including 10–24 years) were included only if data were separately presented for the 10–24-year age group. We included observational studies (surveys, case-control studies, cohort studies), qualitative studies and intervention studies (only if baseline prevalence data were presented). We included studies which examined alcohol use and AUDs defined as per the International Classification of Diseases (ICD)/Diagnostic and Statistical Manual of Mental Disorders (DSM)/clinical criteria or using a standardised screening or diagnostic tool.

### Data

We searched the following databases: PsycARTICLES, PsycInfo, Embase, Global Health, CINAHL, Medline and Indmed. The search strategy was organised under the following concepts: substance (e.g. alcohol, drug), misuse/use disorder (e.g. addiction, intoxication), young people (e.g. adolescent, child) and India (e.g. India, names of individual Indian states). The detailed search strategy is listed in Appendix A.

Two reviewers (DG and KW) independently inspected the titles and abstracts of studies identified through the database search. Any conflicts about eligibility between the two reviewers were resolved by AN. If the title and abstract did not offer enough information, the full paper was retrieved to ascertain whether it was eligible for inclusion. Screening of full texts was done by AN, AG and DG; and any conflicts about eligibility were resolved by UB. Screening of the results of the search was done using Covidence (https://www.covidence.org/), an online screening and data extraction tool.

AN searched the following resources to identify relevant grey literature: Open Grey, OAlster, Google, ProQuest, official English language websites of the World Health Organization and World Bank, English language websites of ministries of each state and union territory within India responsible for substance misuse as well as the official websites of the Indian Narcotics Control Bureau and Ministry of Social Justice and Empowerment.

Any grey literature with relevant data published by a recognised non-governmental organisation, state, national or international organisation was included. Studies were included based on the robustness of study design and quality of data. If there were multiple editions of any published piece of grey literature, only the latest published edition of that report was included. Once retrieved, their titles, content pages and summaries were read by AN and if deemed eligible they were added to a list of potentially eligible reports. If the grey literature's summary, content and title did not include enough information, then the full text was examined by AN to determine eligibility for inclusion.

Finally, experts in the field of substance use disorders in India were contacted to explore if they could identify any further useful sources of information and were invited to submit unpublished data and unreported outcomes for possible inclusion into the review. Reference lists of selected studies, grey literature and relevant reviews were inspected for additional potential studies.

A formal data extraction worksheet was designed to extract data relevant to the study aims. The following data were extracted: centre (e.g. name of city), sampling technique, sample (e.g. general population), sample size, age(s), tool used to measure alcohol use and/or AUD, definitions of alcohol use and AUD, prevalence of alcohol use and/or AUD, age of initiation, type of alcohol, quantity and frequency of alcohol use, attitudes towards alcohol use, effect of alcohol on health, social, educational and other domains, and risk factors/correlates of alcohol use and or AUD. Following Preferred Reporting Items for Systematic Reviews and Meta-Analyses (PRISMA) guidelines (Moher *et al*., 2015), a record was made of the number of papers retrieved, the number of papers excluded and the reasons for their exclusion. AT independently performed data extraction, AG checked the data extraction, and AN arbitrated any unresolved issues. The quality of reporting of included studies was examined using the STROBE Statement – checklist of items that should be included in reports of observational studies (Von Elm *et al*., 2007).

A descriptive analysis of the data was conducted, and the results are mainly reported in a narrative format focusing on each of the objectives described above (Popay *et al*., 2006).

## Results

**Fig. 1.**
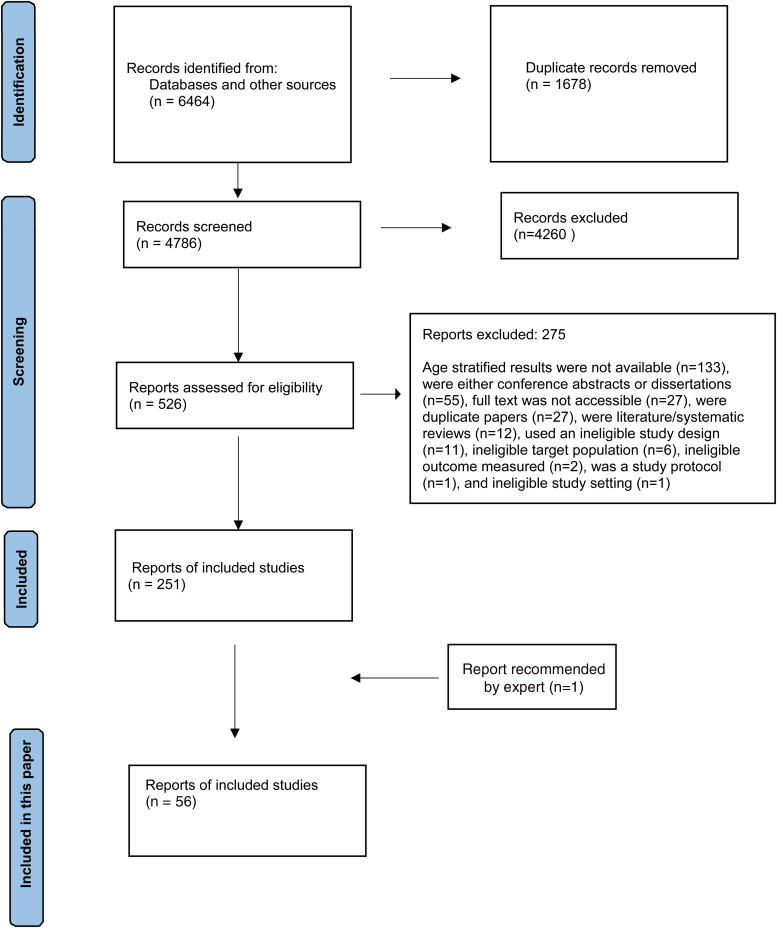
PRISMA flow diagram.

In total, 6464 references were identified through the search strategies described above. Overall, 251 records were eligible for the wider review, of which 55 were about alcohol use and have been reported in this paper (Fig. 1). Additionally, one report of magnitude of substance use in India which was recommended by an expert was also included (Ambekar *et al*., 2019).

### Study descriptions

One study was conducted online (Gupta *et al*., 2018) and one in a national treatment centre in North India (Mandal *et al*., 2019), both of which potentially had access to participants from across the country (Table 1). All the rest were conducted at a single or multiple settings in a city, town, district, village or state. The sample size of the studies ranged from 23 (Bhad *et al*., 2017) to 7350 (Jaisoorya *et al*., 2016). In studies that reported mean age of the samples, it ranged from 13.10 years (Pillai *et al*., 2008) to 20.56 years (Garg *et al*., 2009).

**Table 1:** Description of studies included in the review

Author	Centre	Sampling technique	Sample	*N*	Setting	Age
Ahmad *et al*. (2009)	Aligarh	Probability proportionate to size (PPS)	Male (M), 10–19 y	390	Schools	10–13 (42%), 14–15 (35.6%), 16–19 (22%)
Ambekar *et al*. (2019)	India	Respondent driven sampling (RDS)	10–17 y	NA	Community	10–17 y
Anandi *et al*. (2018)	Kalaburagi	Random	Medical students	224	Medical school	Median 20–21; range 18–23
Armstrong *et al*. (2013)	Delhi	Time location sampling (TLS)	⩾18 y, had injected drugs at least once in past month, not currently enrolled in opioid substitution therapy	420	Needle and syringe programme centres	18–24 (11.3%)
Arya *et al*. (2016)	Haryana	Entire sampling frame (ESF)	Substance users, 12–19 y	89	Drug abuse treatment centre	Mean (s.d.) 17.9 (1.41)
Bahl and Kumari (2016)	Jammu	ESF from randomly selected schools	Students, 9th–12th class	440	Private and public sector high schools	Range 12–19
Bardhan *et al*. (2015)	Guwahati	Cluster sampling	10–19 y	414	Slums of a large city	Mean (s.d.) 14.06 (2.68)
Bashir *et al*. (2015)	Srinagar	Not specified (NS)	Substance use disorder patients, M	125	Drug deaddiction centres	10–19 (16%)
Bhad *et al*. (2017)	Delhi	Consecutive	Inhalant use in past month, M	23	Tertiary drug dependence treatment centre, urban (U)	Range 13–18, mean (s.d.) 16 (1.8)
Deb *et al*. (2013)	Delhi	Random	Ever smokers	300		Range 17–24
Gaidhane *et al*. (2008)	Mumbai	Simple random	Adolescent street children, M	163	U	Range 11–19, median 16
Garg *et al*. (2009)	Chandigarh, Patiala, Amritsar	Convenience	M, undergraduate medical students	152	Medical colleges	Mean (s.d.) 20.56 (1.34)
Gupta *et al*. (2018)	All India	Convenience	13–25 y, lived in India or Australia in past 12 months	330 in India	Online survey	Median 20
Gupta *et al*. (1987)	Ludhiana	Simple random selection of factory workers and rickshaw-pullers. ESF for railway coolies	Youth not in education	257	Workplaces	Range 15–24
Haorongbam *et al*. (2018)	Karnataka	ESF	Medical students	428	Private medical college	Range 17–26, 17–20 (54%), 21–23 (40.4%)
Jain *et al*. (2012)	Mangalore	Random	Students, 13–15 y	413	High schools	Mean (s.d.) 13.72 (0.8)
Jaisoorya *et al*. (2016)	Ernakulam district	Cluster random	Students, classes 8, 10 and 12	7350	Government schools, government-aided schools	Range 12–19, M (s.d.) 15.3 (1.7)
Jayakrishnan *et al*. (2011)	Kerala	ESF from random sampling of schools	Students, 13–19 y	1473	High schools	Mean 15.4 (s.d. 1.5)
Jayakrishnan *et al*. (2016)	Thiruvananthapuram district	ESF from random sampling of schools from random sampling of educational sub-district	M, classes 9–12	1114	Government schools, rural (R)	
Kalpana and Kavya (2012)	Bangalore	NS	College students, 17–21 y	293	Colleges	
Katyal *et al*. (2014)	Meerut	ESF from randomly sampled families	M, ⩾15 y	324	U, slum	15–24 (34.9%)
Kokiwar and Jogdand (2011)	Karimnagar district	Simple random	M, 10–19 y	260	U, slum	Mean (s.d.) 15.03 (3.0)
Kotwal *et al*. (2005)	New Delhi	Random sample of students from random sampling of schools	Students, classes 9 and 11	596	Government and public school	⩽14 (41.28%), 15–17 (58.72%)
Kumar *et al*. (2016)	Jammu	ESF from randomly sampled schools	M, students, 15–19 y	848	Government and private schools	U mean (s.d.) 16.5 (1.55), R mean 16.4 (1.65)
Kundapur and Kodyalamoole (2016)	Mangalore	Random	Medical students	180	Medical colleges	22–23
Lal and Singh (1979)	Punjab	Systematic random	⩾10 y	497	R	10–19 (*n* = 145)
Mahanta *et al*. (2016)	Assam	Convenience	School and college students, 12–20 y	1285	Industrial town	12 (*n* = 1), 13 (*n* = 26), 14 (*n* = 245), 15 (*n* = 296), 16 (*n* = 277), 17 (*n* = 297), 18 (*n* = 128), 19 (*n* = 11), 20 (*n* = 4)
Mandal *et al*. (2019)	Northern India	ESF	Female (F), substance users	28	Tertiary addiction treatment centre	Mean (s.d.) = 15.89 (2.72)
Medhi *et al*. (2006)	Dibrugarh district	Random sampling of households from randomly sampled tea plantations	Tea plantation youths, 15–24 y	650	Households	Mean (s.d.) = 19.3 (2.8)
Meshram *et al*. (2015)	Hyderabad	Convenience	Street children, 8–18 y	305	Streets	Mean (s.d.) 14.5 (2.4), 14–18 (69.8%)
Mohan *et al*. (1981)	New Delhi	Stratified cluster	M, senior high school students	254	High schools	Range 12–18
Mohan *et al*. (1978b)	Punjab	Random to select community development blocks and villages. Households selected by systematic sample with random start	M, 15–19 y	281	R	
Mohan *et al*. (1978a)	Delhi	ESF	College students	502	Colleges	16 (*n* = 66), 17 (*n* = 52), 18 (*n* = 120), 19 (*n* = 100), 20 (*n* = 30), ⩾21 (*n* = 14)
Mohan and Arora (1976)	Delhi	ESF	M, university students	1144	Universities	
Mohanan *et al*. (2014)	Udupi district	Schools selected randomly, students enrolled systematically	School students, 15–19 y	376	Schools and colleges	Mean M 17.09, F 16.09
Mohanty *et al*. (2013)	Berhampur	NS	Students	720	Professional colleges in major trade/educational city	Range 18–20+
Nadkarni *et al*. (2015)	Goa	ESF	16–24 y	3663	Rural farming and urban administrative	Mean (s.d.) 19.5 (2.5)
Nagendra and Koppad (2017)	Shivamogga	Multistage random	10–19 y	480	U, R	U 10–14 (36%), R 10–14 (73%), U 15–19 (64%), R 15–19 (27%)
Naik *et al*. (2011)	Mumbai	Universal sampling	Street children	217	Shelter homes	Range 10–18, mean M 13.96, F 13.91
Ningombam *et al*. (2011)	Imphal	Random	Higher secondary students, classes 11 and 12	1012	Government and private higher secondary schools	Range 15–22, median 17
Nuken *et al*. (2013)	Nagaland	Respondent driven	Female sex workers, ⩾18 y	417	NS	40.6% <25 yrs
Olumide *et al*. (2014)	Delhi	Respondent driven	Adolescents in vulnerable neighbourhoods	2332	Disadvantaged settings	Range 15–19, 15–16 (47.5%)
Pillai *et al*. (2009)	Goa	ESF	16–24 y	3662	Rural (R) farming, urban (U) commercial communities	Mean (s.d.) 19.4 (2.5)
Pillai *et al*. (2008)	Goa	ESF	12–16 y	2048	As above	Mean 13.49 (urban); 13.10 (rural)
Rani and Sathiyaskaran (2013)	Chennai	Random	Students, 12–18 y	1842	Private and public sector high schools	12–14 (41.3%)
Rathore *et al*. (2015)	Rajasthan	ESF	Medical and dental students	510	Medical and dental colleges	
Sandhya *et al*. (2013)	Mangalore	Convenience	Pre university college students	200	Pre university college	Mean (s.d.) 16.7 (0.5)
Sarangi *et al*. (2008)	Sambalpur	Ward selected by simple random, each house surveyed until sample reached	Adolescents	502	U, slum	
Saxena *et al*. (2010)	Doiwala	ESF of students from random sampling of colleges	M, 10–12th classes	511	Intermediate schools	Range 14–19
Sharma *et al*. (2016)	New Delhi	Random	M, under enquiry	487	Observation home for juveniles under enquiry	Range 8–18, mean (s.d.) 14.17 (1.57)
Sharma *et al*. (2015)	Delhi	Random	M, 8–18 y	487	Juvenile home	Mean 14.17
Shrivastava and Bobhate (2010)	Mumbai	Universal sampling	Ever tobacco users, 15–24 y	133	Urban health centre	15–18 (23.3%), 18–21 (42.1%), 21–24 (34.6%)
Singh *et al*. (2010)	Mumbai	Cluster sampling	M, 18–29 y	1239	Low-income communities	18–20 (*n* = 161), 21–24 (*n* = 340)
Singh and Preet (1981)	Patiala	NS	Students	444	Schools and colleges	Range 12–20
Sukhwal and Suman (2013)	Bangalore	NS	College students	236	English medium colleges	Range 18–21
Tsering *et al*. (2010)	West Bengal	Multistage random	High school students	416	High schools	Mean (s.d.) 15 (3.67)

### Prevalence of alcohol use and AUD

#### Ever use

The prevalence of ever use or lifetime use, broadly defined as consumption of alcohol at least once in their lifetime, ranged from 3.9% in school students aged 12–18 years (Rani and Sathiyaskaran, 2013) to 69.8% in 22–23-year-old medical students (Kundapur and Kodyalamoole, 2016) (Table 2). Ever use in females ranged from 6.5% in students from class 8 to class 12 (age 12–19 years) (Jaisoorya *et al*., 2016) to 52% in an online survey of adolescents aged 13–17 years (Gupta *et al*., 2018), and in males it ranged from 9.79% in students from classes 9 and 11 (age up to 17 years) (Kotwal *et al*., 2005) to 47% in an online survey of adolescents aged 13–17 years (Gupta *et al*., 2018). The prevalence of ever use in rural areas ranged from 7.37% in high school students (Tsering *et al*., 2010) to 20% in students aged 15–19 years (Kumar *et al*., 2016), and in urban areas it ranged from 5.23% in high school students (Tsering *et al*., 2010) to 23.08% in students aged 15–19 years (Kumar *et al*., 2016).

**Table 2:** Prevalence of alcohol use and alcohol use disorders

Author, year	Standardised tool	Definition of alcohol use	Prevalence of alcohol use	Definition of alcohol use disorder (AUD)	Prevalence of AUD
Ahmad *et al*. (2009)			3.8% of any substance users		
Ambekar *et al*. (2019)		Current alcohol use	1.3%		
Anandi *et al*. (2018)	AUDIT		25.4%	>13 in F or >15 in M = alcohol dependence Binge drinking = ⩾5 alcoholic drinks in M or ⩾4 in F on the same day on at least 1 day in the past month	Among drinkers: hazardous drinking = 19.29%; alcohol dependence = 8.7%; binge drinking = 14%
Armstrong *et al*. (2013)		Ever user Current user (past month)	Ever user but not in past month = 43.4% (95% CI 36.1–50.7%) Current user = 35.6%		
Arya *et al*. (2016)			35%		
Bahl and Kumari (2016)		Ever use = tried anytime	Ever use = 22.0% urban, 15.6% rural. Current use = 2.9% urban, 4.6% rural		
Bardhan *et al*. (2015)		Current user = taken substance more than 10 times in lifetime and used in past month	Alcohol use among current substance users: 14.5%; 21.2% M, 0% F		
Bashir *et al*. (2015)					36.8% of patients seeking treatment for substance use disorders
Bhad *et al*. (2017)			Ever use 34.8% Current use 21.7%		
Deb *et al*. (2013)		Alcohol use among smokers	88.0% M, 74.0% F		
Gaidhane *et al*. (2008)				One or more of following occurs in 12 months: recurrent substance use results in failure to fulfil major role obligations at work or home, leads to physically hazardous situation, or causes legal problems	37%
Garg *et al*. (2009)	ICD 10 CAGE	Ever use Past year use	Ever use = 56.57% Past year use = 53.94%		Of those who used in past year, 6.09% dependent, 41.46% problem drinkers
Gupta *et al*. (2018)		Lifetime use = consumed ever in lifetime, recent use = any use in past 4 weeks	Lifetime use: 47% M 52% F Recent use: 21% M 21% F		
Gupta *et al*. (1987)		Ever user = ever used even once. Recent user = used in past 12 months. Current user = past month use but not daily or weekly	51.3% ever used 48.6% recent use, 27.6% current use		
Haorongbam *et al*. (2018)	AUDIT	Consumed alcohol at least once	49.1%	Dependence = AUDIT score ≥20	3.7% dependence
Jain *et al*. (2012)			4.6%		
Jaisoorya *et al*. (2016)	ASSIST	Lifetime user = ever used any alcohol	Lifetime use: 15% overall, 23.2% M, 6.5% F	Dependent user = alcohol involvement score 27+	Among lifetime users, 1.6% are dependent (2% males, 0% females)
Jayakrishnan *et al*. (2016)		Ever user in current academic year = used at all during the academic time period during which study was conducted Current user = using alcohol three or more days a week	Ever use in past year = 5.6% (95% CI 4.25–6.95) Current use = 0.8%		
Jayakrishnan *et al*. (2011)			0.5% current use of alcohol		
Katyal *et al*. (2014)	AUDIT	Current drinker = had alcohol in past year Teetotaller = 0 on AUDIT	Of 15–24 yrs, 43.9% were teetotallers, 13.5% current drinkers		
Kokiwar and Jogdand (2011)			12.9% of substance users used alcohol		
Kotwal *et al*. (2005)			Ever use: 9.23% (98% CI 6.67–12.35), 8.55% F, 9.79% M		
Kumar *et al*. (2016)		Ever use	28.08% urban, 20% rural		
Kundapur and Kodyalamoole (2016)		Ever user = used substance once or more in life	69.8%	Dependence = consumers have tolerance, withdrawal symptoms, persistent desire to cut down drinking. Great amount of time of consumers is spent with activity related to alcohol. Social, occupational or recreational activities are given up by dependents, continued use despite of knowledge of serious social, psychological and physical problems Binge drinker: consume 5+ drinks in single sitting for man or 4+ drinks in single sitting for woman	Of consumers, 30% were binge drinkers, 10% alcohol dependent
Mahanta *et al*. (2016)		User = all students who ever had commercial or homemade alcoholic drink in past	39.8%		
Mandal *et al*. (2019)			28.6% used ever, 28.6% in last year, 21.4% in last month		10.7% dependent ever
Medhi *et al*. (2006)		Used regularly for at least one month	32.2%; 43.9% M, 24.6% F		
Meshram *et al*. (2015)			12–18 yrs: 17% were consuming alcohol		
Mohan *et al*. (1981)		Current use = use over past year, lifetime user = used ever	Current user = 10.6%, lifetime user = 16.1%		
Mohan *et al*. (1978b)		Current use = use over past year	32.9%		
Mohan *et al*. (1978a)		Use = use of substance without medical prescription	23.5% overall, 35.9% males, 10.6% females		
Mohan and Arora (1976)		Use of substance without medical prescription	18.58%		
Mohanan *et al*. (2014)		Use of alcohol in past 6 months	5.85% (95% CI 3.80–8.50)		
Mohanty *et al*. (2013)			Of substance users, 53.6% take alcohol		
Nagendra and Koppad (2017)			Drinkers: 2.08% urban, 1.25% rural		
Naik *et al*. (2011)			23.9% use alcohol, 37.7% M, 0% F		
Ningombam *et al*. (2011)		Ever user = use at least once in lifetime, recent use = within past 12 months, current = within past 30 days	Ever use = 29%, recent use = 15%, current use = 7%		
Nuken *et al*. (2013)		Daily alcohol use = daily consumption of alcohol in past 1 month	11.3%		
Olumide *et al*. (2014)		Ever use = use ever in lifetime, current use = use in past 30 days	Ever use: 6.7%, current use = 2.1%		
Pillai *et al*. (2009)		Used in past 3 months	5.4%		
Pillai *et al*. (2008)		Used in past 12 months	1.8%		
Rani and Sathiyaskaran (2013)		Ever user = used at least once	3.9%		
Rathore *et al*. (2015)			10.74% among <20 yrs		
Sandhya *et al*. (2013)	Comprehensive effects of alcohol questionnaire	Ever consumed alcohol	19.5%; 25% M, 14% F		
Sarangi *et al*. (2008)			14.7% of substance users used alcohol		
Saxena *et al*. (2010)		Alcohol use amongst those with substance abuse	8.7%		
Sharma *et al*. (2016)			13–14 yrs 16.8% 15–16 yrs 15.1% 17–18 yrs 17.9%		
Sharma *et al*. (2015)		Current use = use in last 30 days	Ever use = 6.7%, current use = 2.1%		
Shrivastava and Bobhate (2010)			34.6%; 44.2% M, 0% F		
Singh and Preet (1981)		Ever use = use anytime in life, current use = use at least once in past 30 days	Ever use 25.67%, current use 13.96%		
Tsering *et al*. (2010)		Ever use used at any point in life	7.37% rural, 5.23% urban		

#### Current use

The definition of current use of alcohol varied across studies. The more commonly used definitions were alcohol consumption at *least once in the past year* for which the prevalence ranged from 10.6% in senior high school students aged 12–18 years (Mohan *et al*., 1981) to 32.9% in 15–19-year-old individuals from rural settings (Mohan *et al*., 1978b); and *at least once in the past 30 days (month)* for which the prevalence ranged from 2.1% (Sharma *et al*., 2015) in 15–19-year olds from disadvantaged urban settings and 35.6% in injectable drug users attending needle and syringe programme centres (Armstrong *et al*., 2013). Some studies did not define current use and others used non-standard definition of current use such as ‘who had not used drugs either daily or weekly in the past month’ (27.6%) (Gupta *et al*., 1987), and ‘habit of using alcohol, 3 days or more a week’ (0.8%) (Jayakrishnan *et al*., 2016). The biggest countrywide survey of substance use in India reported a prevalence of current alcohol use to be 1.3% amongst those aged 10–17 years (Ambekar *et al*., 2019).

#### AUD

Some studies reported the prevalence of AUDs and defined them using standardised tools (Alcohol Use Disorder Identification Test [AUDIT], CAGE questionnaire, Alcohol, Smoking and Substance Involvement Screening Test [ASSIST]), ICD 10 criteria or bespoke definitions. Among medical students (18–23 years) who were drinkers, the prevalence of hazardous drinking was 19.29% (Anandi *et al*., 2018), alcohol dependence was 3.7–10% (Kundapur and Kodyalamoole, 2016; Haorongbam *et al*., 2018), binge drinking 14–30% (Kundapur and Kodyalamoole, 2016; Anandi *et al*., 2018) and ‘problem drinking’ (not defined) was 41.46% (Garg *et al*., 2009). Among students of classes 8, 10 and 12 (12–19 years), 1.6% (2% males, 0% females) of lifetime users had alcohol dependence (Jaisoorya *et al*., 2016). In adolescent street children (11–19 years), 37% had AUD defined as recurrent substance use resulting in one or more of the following occurring in 12 months: failure to fulfil major role obligations at work or home leads to a physically hazardous situation, or causes legal problems (Gaidhane *et al*., 2008).

### Patterns of drinking

Among drinkers, 0.6–10.4% consumed every day (Armstrong *et al*., 2013; Jaisoorya *et al*., 2016; Kundapur and Kodyalamoole, 2016), 19.1–40% consumed at least once a week (Armstrong *et al*., 2013; Kundapur and Kodyalamoole, 2016), 3.8% consumed weekly (Jaisoorya *et al*., 2016), 9.5% consumed less than once a week (Armstrong *et al*., 2013) and 10.6% consumed monthly (Jaisoorya *et al*., 2016) (Table 3). Usual median number of drinks consumed among those between 13 and 17 years was 3.5 for both males and females (Gupta *et al*., 2018). Among 10–19-year-old males from an urban slum over the past month, 54.2% consumed up to 50 ‘pegs’ of alcohol (Kokiwar and Jogdand, 2011). Among males from a low-income community, in those between 18 and 20 years, 88.2% were ‘low drinking’ (low amount/low frequency, low amount/moderate frequency or substantial amount/low frequency), 9.3% were moderate drinking (low amount/high frequency or substantial amount/moderate frequency) and 2.5% were high drinking (substantial amount/high frequency); and in those between 20 and 24 years, 82.6% were low drinking, 13.5% were moderate drinking and 3.8% were high drinking (Singh *et al*., 2010).

**Table 3:** Initiation of, attitudes towards, patterns of and correlates of drinking

Author	Age of initiation	Frequency/quantity	Attitudes	Risk factors/correlates
Anandi *et al*. (2018)				Males more likely than females to experiment with alcohol at least once (*p* < 0.001)
Armstrong *et al*. (2013)		Less than once a week = 9.5% (95% CI 6.9–12.1%). At least once a week = 19.1 (95% CI 14.4–23.7%). Every day = 7.0% (95% CI 3.3–10.7%)		
Bashir *et al*. (2015)	41.3% initiated between 10 and 19 yrs			
Garg *et al*. (2009)	Mean age at first drink: 18.3 yrs, s.d. = 1.98			
Gupta *et al*. (2018)	Mean = 16.5 yrs, s.d. = 3.5	Usual median drinks = 3.5 (IQR = 2.0–4.5) for males 13–17 yrs. Usual median drinks = 3.5 (IQR = 1.75–5.5) for females 13–17 yrs		Higher usual consumption associated with increased age (*p* = 0.003), sharing own alcohol-related content on Twitter (*p* = 0.003) Lower usual consumption associated with: friends sharing less alcohol-related information on Facebook (*p* < 0.001), YouTube (*p* < 0.001)
Haorongbam *et al*. (2018)			17.8% use drinking alcohol as coping mechanism for depression symptoms. Reasons for initiation: 15.2% friends' influence, 17.5% party, 9.8% social gathering, 19.6% curiosity, 3.3% others. Reason for drinking: 31.5% enjoyment, 9.6% to take mind off other issues, 14.8% socialisation	
Jain *et al*. (2012)		3 of 19 drinkers consumed more than 60 ml once a week		Drinkers more likely from private schools than public schools (8.4% *v*. 1%; *p* = 0.001)
Jaisoorya *et al*. (2016)		Among users, monthly: 10.6%, 10.5% M, 3.8% F Weekly: 3.8%, 3.8% M, 0.9% F Daily: 0.6%, 0.7% M, 0% F		Male: OR = 2.8 (95% CI 2.3–3.4) Older age: OR = 1.15 (95% CI 1.05–1.25) Having part-time job: OR = 1.4 (95% CI 1.1–1.8) Failed subject in school: OR = 1.2 (95% CI 1.01–1.4) Failed year in school: OR = 1.4 (95% CI 1.1–1.8) Tobacco use: OR = 8.1 (95% CI 6.4–10.2) Illicit drug use: OR = 2.5 (95% CI 1.7–3.7) Suicidal thoughts: OR = 1.7 (95% CI 1.4–2.1) ADHD symptoms: OR = 1.02 (95% CI 1.01–1.03) Non-contact sexual abuse: adjusted OR = 2.2 (95% CI 1.7–2.7)
Jayakrishnan *et al*. (2016)				Alcohol use in household: 55.4% ever users *v*. 33.2% never users, *p* = 0.0001
Kalpana and Kavya (2012)			Knowledge of harmful effects of alcohol Total: 55.3% no risk, males: 43.4% thought no risk, females: 69.4% no risk Village: 64.4% no risk Town: 60.7% thought no risk City: 50.0% no risk	
Kokiwar and Jogdand (2011)		In the last month, 54.2% consumed up to 50 pegs of alcohol		
Kumar *et al*. (2016)	Age at which first drink of alcohol taken (among ever users): Urban: 6.50% ≤ 8 yrs, 8.94% 9–10, 27.65% 11–12, 26.83% 13–14, 24.39% 15–16, 5.69% ≥ 17 Rural: 6.10% ≤ 8 yrs, 10.98% 9–10, 39.02% 11–12, 30.49% 13–14, 10.98% 15–16, 2.44% ≥ 17			
Kundapur and Kodyalamoole (2016)	25.6% students started consuming at 15–17 yrs, 10.4% started <15 yrs	Among users: 10.4% consumed every day, 16.70% twice a week, 40% once a week 16.9% drink <5 pegs, 30% drink 4–5 pegs, 35.4% drink 2–3 pegs	44% consider it safe to consume alcohol, 88% believe drinking patterns are mood dependent	83.3% males *v*. 38% females, *p* < 0.005
Lal and Singh (1979)	52.7% initiated 10–19 yrs			
Mahanta *et al*. (2016)	Commercially available alcohol: mean = 13.90, s.d. = 2.194 Locally brewed alcohol: mean = 11.09, s.d. = 2.775			
Mandal *et al*. (2019)	Mean = 15.28, s.d. = 1.77			Commercially available alcohol: >15 yrs OR = 2.226, 95% CI 1.475–3.357 Male OR = 2.701, 95% CI 1.782–4.094 Close friends take any substance OR = 2.348, 95% CI 1.580–3.488 Close friends forces OR = 2.064, 95% CI 1.199–3.551 Father alcohol and tobacco user OR = 2.374, 95% CI 1.372–4.110 Locally brewed alcohol: >15 yrs OR = 0.76, 95% CI 0.58–0.99 Rural OR = 1.66, 95% CI 1.26–2.19 Close friends take any substance OR = 1.39, 95% CI 1.04–1.88 Close friends forces OR = 1.69, 95% CI 1.02–2.81 Father alcohol and tobacco user OR = 1.95, 95% CI 1.34–2.84 Mother alcohol user OR = 5.17, 95% CI 2.84–9.42 Mother tobacco user OR = 2.02, 95% CI 1.48–2.78 Mother alcohol and tobacco user OR = 4.60, 95% CI 3.21–6.59
Medhi *et al*. (2006)				41.8% among 20–24 yrs *v*. 21.9% of 15–19 yrs, *p* < 0.01 43.9% males *v*. 24.6% females, *p* < 0.01 42.0% unmarried, 26.2% married, *p* < 0.01
Mohan *et al*. (1978b)		Of users: 3.0% regular user		Education status: see prevalence data, *p* < 0.01
Mohan and Arora (1976)		2.35% casual, 16.23% moderate to heavy		
Mohanan *et al*. (2014)				Male: OR = 4.82, 95% CI 1.3–17.5 Alcohol habit among family OR = 6.23, 95% CI 3.45–8.95
Mohanty *et al*. (2013)	18.0% of users initiated 10–14 yrs, 55.1% initiated 15–19 yrs, 26.9% 20 + yrs			
Nadkarni *et al*. (2015)				Perpetuation of physical violence by men OR = 2.37 (95% CI 1.69–3.31, *p* < 0.001)
Nagendra and Koppad (2017)	Urban = 12.5, s.d. = 3.57; rural = 10.66, s.d. = 4.02; *p* < 0.0001			
Olumide *et al*. (2014)	Mean age of first glass of alcohol = 14.4, s.d. = 3.4	27.9% used once or twice a week, 1.5% once per week, 1.5% more than once a week 4.8% ever taken 5+ drinks in 2 h		
Pillai *et al*. (2009)				Suicidal thinking/planning/attempts, OR = 2.7 (95% CI 1.7–4.4, *p* < 0.05)
Rani and Sathiyaskaran (2013)				Parent/guardian who drinks alcohol, OR = 2.66 (95% CI 1.66–4.28, *p* < 0.001) Lack of parental supervision, OR = 1.87 (95% CI 1.17–3.00, *p* < 0.05) Not having understanding parents OR = 2.45 (95% CI 1.44–4.18, *p* < 0.001) Ever use: 5% private school students *v*. 2.4% public school students (*p* < 0.01)
Rathore *et al*. (2015)	Overall: 4.26% of users initiated <12 yrs, 19.15% 12–18, 76.60% >18 Males: 5.88% of users initiated <12 yrs, 16.18% 12–18, 77.94% >18 Females: 0% of users initiated <12 yrs, 26.92% 12–18, 73.08% >18			10.74% among <20 yrs, 18.45% among 21–25, 40.38% among 26–30, *p* < 0.001
Sandhya *et al*. (2013)			Stronger endorsement of negative reinforcements (e.g. cognitive impairment, risk taking) than of possible positive reinforcements (e.g. sociability, tension reduction). Females felt alcohol consumption could not reduce tension and males thought it could (*p* < 0.001). Females also endorsed increased sociability and cognitive impairment compared to males (*p* < 0.005)	
Sarangi *et al*. (2008)	Mean = 16, s.d. = 1.5			
Sharma *et al*. (2015)	Age first finished a glass of alcohol: mean = 14.4 yrs, s.d. = 3.4	In last 30 days: 66.2% used not at all, 27.9% once or twice, 1.5% once a week, 1.5% more than once a week, 0 every day, 2.9% don't know, 4.8% ever taken 5+ drinks in 2 h		
Singh *et al*. (2010)		Among 20 and below: 88.2% low amount/low frequency, low amount/moderate frequency, and substantial amount/low frequency (low drinking), 9.3% low amount/high frequency and substantial amount/moderate frequency (moderate), 2.5% substantial amount/high frequency (high) 21–24 yrs: 82.6% low drinking, 13.5% moderate, 3.8% high		
Sukhwal and Suman (2013)				Higher acceptance of alcohol is associated with lower spirituality, less religiosity, less God Consciousness and less formal religious practices
Tsering *et al*. (2010)			Knowledge of harm of alcohol among substance users: 61.5% urban, 30.8% rural	

### Initiation age

The mean age for initiation of drinking ranged from 14.4 to 18.3 years (Table 3). The mean age of initiation was significantly lower in rural areas compared to urban areas [10.66 (s.d. 4.02) *v*. 12.5 (s.d. 3.57); *p* < 0.0001] (Nagendra and Koppad, 2017); and locally brewed alcohol [mean (s.d.) 11.09 (2.775)] was initiated at a younger age compared to commercially available alcohol in an industrial town [mean (s.d.) 13.90 (2.194)] (Mahanta *et al*., 2016).

Among male substance use disorder patients at drug deaddiction centres, 41.3% had initiated alcohol use between 10 and 19 years (Bashir *et al*., 2015). Among 22–23-year-old medical students, 25.6% had started consuming alcohol between 15 and 17 years, and 10.4% had started consuming alcohol before they were 15 years (Kundapur and Kodyalamoole, 2016).

In students between 18 and 22 years, 18.0% had initiated drinking between 10 and 14 years, 55.1% had initiated between 15 and 19 years, and 26.9% after 19 years (Mohanty *et al*., 2013). Among medical and dental students, 4.26% initiated before 12 years, 19.15% initiated between 12 and 18 years, and 76.60% initiated after 18 years (Rathore *et al*., 2015). Comparing males and females, 5.88% males (*v*. 0% females) initiated before 12 years, 16.18% (*v*. 26.92%) initiated between 12 and 18 years, and 77.94% (*v*. 73.08%) initiated after 18 years (Rathore *et al*., 2015). Finally, comparing urban and rural drinkers, 6.50% urban drinkers (*v*. 6.10% rural) initiated before 8 years, 8.94% (*v*. 10.98%) initiated between 9 and 10 years, 27.65% (*v*. 39.02%) initiated between 11 and 12 years, 26.83% (*v*. 30.49%) initiated between 12 and 14 years, 24.39% (*v*. 10.98%) initiated between 15 and 16 years, and 5.69% (*v*. 2.44%) initiated after 17 years (Kumar *et al*., 2016).

### Knowledge and attitudes

Overall, 55.3% of college-going students (17–21 years) believed that there was no risk of harmful effects of alcohol; with more females than males who believed that there was no risk (69.4% *v*. 43.4%); and a higher proportion from villages (64.4%) thought there was no risk as compared to those from towns (60.7%) or cities (50.0%) (Kalpana and Kavya, 2012) (Table 3). Among medical students (22–23 years), 44% considered it safe to consume alcohol, and 88% believe drinking patterns are mood-dependent (Kundapur and Kodyalamoole, 2016).

In medical students (17–23 years), reasons for initiation of drinking included curiosity (19.6%), attending a party (17.5%), friends' influence (15.2%) and social gatherings (9.8%); and reasons for continued use included enjoyment (31.5%), as a coping mechanism for depressive symptoms (17.8%), socialisation (14.8%) and to take mind off other issues (9.6%) (Haorongbam *et al*., 2018). Among college-going students (mean age 16.7 years; s.d. 0.5) there was a stronger endorsement of negative reinforcements (e.g. cognitive impairment, risk taking) than of possible positive reinforcements (e.g. sociability, tension reduction); and compared to males, significantly more females felt alcohol consumption could not reduce tension and endorsed increased sociability and cognitive impairment (Sandhya *et al*., 2013). Knowledge of harm of alcohol among substance users was greater in adolescents from urban than rural areas (61.5% *v*. 30.8%) (Tsering *et al*., 2010).

#### Risk factors/correlates

The cross-sectional nature of the studies only allowed the examination of correlates of alcohol use (Table 3). Alcohol consumption was associated with being male (Medhi *et al*., 2006; Mohanan *et al*., 2014; Jaisoorya *et al*., 2016; Kundapur and Kodyalamoole, 2016; Anandi *et al*., 2018; Mandal *et al*., 2019), older age (Medhi *et al*., 2006; Rathore *et al*., 2015; Jaisoorya *et al*., 2016; Gupta *et al*., 2018; Mandal *et al*., 2019) and going to private rather than public schools (Jain *et al*., 2012; Rani and Sathiyaskaran, 2013). Specifically for locally brewed alcohol, it was associated with younger age and rural residence (Mandal *et al*., 2019). Alcohol consumption was associated with having a part-time job, and failing a subject or a year in school (Jaisoorya *et al*., 2016).

Alcohol use in adolescents was associated with parental/guardian's use of alcohol or tobacco, lack of parental supervision, and not having ‘understanding’ parents (Rani and Sathiyaskaran, 2013; Mohanan *et al*., 2014; Jayakrishnan *et al*., 2016; Mandal *et al*., 2019). Alcohol use decreased with a decrease in the frequency of friends sharing alcohol-related information on Facebook and YouTube; and increased frequency of sharing personal alcohol-related content on Twitter was associated with an increase in alcohol use (Gupta *et al*., 2018). Alcohol consumption was also associated with close friends using substances (any type) or peer pressure to drink alcohol (Mandal *et al*., 2019).

Alcohol consumption was associated with tobacco use, illicit drug use, attention deficit hyperactivity disorder (ADHD) symptoms, suicidal thinking, planning and attempts, and non-contact sexual abuse and perpetuation of violence (Nadkarni *et al*., 2015; Jaisoorya *et al*., 2016). Finally, higher acceptance of alcohol is associated with lower spirituality, less religiosity, less ‘God Consciousness’ and less formal religious practices (Sukhwal and Suman, 2013).

#### Quality of reporting studies

In 42 of the 57 studies, there was appropriate reporting of more than 70% of the 22 STROBE criteria (Appendix B). Only one study reported on all the 22 criteria (Nadkarni *et al*., 2015). For 15 of the 22 criteria, there was appropriate reporting in more than 70% of the studies. The poorest reporting was about study biases, generalisability of the findings, and role of the funder.

## Discussion

The existing evidence base has several limitations which preclude a robust synthesis and any conclusions we draw are, at best, exploratory in nature. Although the information about AUDs is relatively limited, the prevalence among drinkers appears to be high, and the patterns of drinking in a reasonably high proportion were suggestive of risky drinking (heavy drinking that puts the drinker at risk of developing problems), especially considering that this is a young population with a relatively short drinking history.

This is consistent with the steady rise in recorded alcohol consumption in most developing countries, albeit from relatively low base prevalence rates. It also parallels the increases in adult per capita consumption of alcohol and heavy episodic drinking that have been observed in India and other developing economies in east Asia, south Asia and southeast Asia (Shield *et al*., 2020). Amongst adolescents, the prevalence of current alcohol use in Sri Lanka was 3.4% (95% CI 2.6–4.3) (Senanayake *et al*., 2018), lifetime alcohol use in males was 45% (26% risky drinking) in Pakistan (Shahzad *et al*., 2020), alcohol use was reported by 19% from traditional non-alcohol using ethnic groups and 40% from traditional alcohol using ethnic groups in Nepal (Parajuli *et al*., 2015), and 13% in Bhutan (Norbu and Perngparn, 2014).

The data about patterns of drinking observed among adolescents in India are inconclusive but there appears to be some tendency towards heavy drinking. Among adolescents across several countries, there are consistent reports of binge drinking as a social norm among peer groups (Russell-Bennett *et al*., 2010). The prevalence of binge drinking increases from age 15–19 years to the age of 20–24 years, and among drinkers, binge drinking is higher among the 15–19 years age group compared with the total population of drinkers (World Health Organization, 2018). This means that 15–24-year-old current drinkers often drink in heavy drinking sessions, and hence, except for the Eastern Mediterranean Region, the prevalence of such drinking among drinkers is high in adolescents (around 45–55%) (World Health Organization, 2018).

In India, the age of initiation commonly was mid- to late-teens; and male gender, rural residence and locally brewed alcohol were associated with earlier initiation of drinking. Across most of the world, initiation of alcohol use among adolescents takes place at an early age, usually before the age of 15 years. Among 15-year-olds, there is a high prevalence of alcohol use (50–70%) during the past 30 days in many countries of the Americas, Europe and Western Pacific; and the prevalence is relatively lower in African countries (10–30%) (World Health Organization, 2018). However, across the world, there is a huge variation in alcohol use among boys and girls of 15 years of age and vary from 1.2% to 74.0% in boys and 0% to 73.0% in girls (World Health Organization, 2018). Finally, with the strategic targeting of adolescents as alcohol consumers by the industry, increasing overall population prevalence and normalisation of drinking alcohol, and the increasing normalisation by virtue of learning more about how adolescents in other countries drink, one could speculate that the age of initiation would reduce and prevalence of alcohol consumption in adolescents in India would rise, in the coming years.

In India, knowledge about alcohol and its potential harms was limited in rural areas. The reasons for starting and continuing drinking were a mix of expected enhancement of positive experiences and dampening of negative affect. This is consistent with findings in Indian adults where alcohol consumption was seen to be mainly associated with expectations about reduction in psychosocial stress and providing pleasure (Nadkarni *et al*., 2013). Across the world, adolescents primarily report drinking for social motives or enjoyment – enjoyment (Argentina) (Jerez and Coviello, 1998), to make nights out more pleasurable (UK) (Plant *et al*., 1990) and being social (Canada) (Kairouz *et al*., 2002). Coping motives, on the other hand, are less common, but are associated with AUDs later in adulthood (Carpenter and Hasin, 1999). The difference in drinking motives between adolescents from India (a mix of pleasure and coping) and other countries (primarily pleasure), and the similarity between reasons given by Indian adolescents and Indian adults, possibly reflect contextual/cultural differences and will have implications on transferability of interventions from other contexts and wider age-applicability of interventions developed for adults in India.

We can broadly organise our findings about correlates for drinking into socio-demographic characteristics (e.g. age, gender), immediate environment (e.g. parents, friends, digital space) and clinical correlates (e.g. other substance use, suicidal thoughts). Risk and protective factors influencing the use of alcohol in adolescents are both proximal and distal factors and include individual cognitions and peer-influence risk factors (e.g. attitudes favourable to alcohol use and peer drinking), family environment (e.g. parental discipline and family bonding) and school context (e.g. academic commitment and achievement) (Bryant *et al*., 2003; Fisher *et al*., 2007; Patock-Peckham and Morgan-Lopez, 2010). Most commonly adolescent males drink more often than adolescent females, but there has been some blurring of the distinction between the genders in developed countries (Currie *et al*., 2004; Hibell *et al*., 2009). This convergence of drinking patterns is particularly seen in the Nordic countries, Ireland, the UK and the USA, and manifests as almost equal prevalence rates for consumption of spirits and similar frequency of intoxication for both genders (Hibell *et al*., 2009). Evidence from South Asian countries indicates that male gender, age greater than 14 years, depression, religious beliefs, parental/family members' drinking, reduced parental supervision, peer-drinking/pressure/approval and urban neighbourhood are associated with adolescent drinking (Athauda *et al*., 2020).

The most important study finding is that despite several studies over the years, the evidence base has several gaps, notably the limited geographical span, small sample sizes and heterogeneous definitions of alcohol use and AUDs. Of particular importance are the various sample selection strategies, especially for the smaller studies, which limit the generalisability of findings. Another gap is the lack of consistency in the measurement of alcohol use, which is especially critical in a context where ‘standard drink’ does not translate semantically or literally into the vernacular, and there is an immense variability in the types of alcoholic beverages (commercial, licit non-commercial, illicit home-brewed, adulterated alcoholic beverages) and in the type and size of vessels from which alcohol is poured or consumed in. Additionally, there were several gaps in the reporting of many studies which raise questions about their internal validity. In the absence of critical information such as data sources, measurement and statistical methods, it is difficult to draw an inference about the robustness of the studies which had inadequate reporting (Appendix B). Finally, although the cross-sectional design of the studies allows us to examine the prevalence of alcohol use and AUDs, it limits the conclusions that we can draw about causal relationships between the various potential risk factors and alcohol use/AUDs.

Although the included studies are not without limitations that are important to consider before drawing conclusions, this synthesis allows us to get a reasonable understanding of alcohol use among adolescents in India and derive preliminary conclusions that the prevalence is high and rising, which brings with it the attendant burden of the associated adverse impacts. Furthermore, despite the gaps in the available data, it carries several implications for policy makers. Because alcohol is an important cause of motor vehicle accidents and suicide, which are the leading causes of death among adolescents in India (Joshi *et al*., 2017), interventions that seek to help adolescents avoid or better manage alcohol consumption are a priority. Examples of such evidence-based interventions include public health engagement campaigns to increase awareness of alcohol-related harms, advocacy through community engagement/mobilisation to promote better enforcement of laws related to drinking, engagement with alcohol outlets to promote responsible beverage service, and engaging adolescents and families including through peer-led classroom curriculum to enhance the resilience of adolescents, improve family socialisation and increase awareness of alcohol-related harms (McLeroy *et al*., 2003; Hawkins *et al*., 2008; Wakefield, Loken, and Hornik, 2010; Hallgren and Andréasson, 2013). The most important implication of our review, however, is the need to develop the very nascent literature base through robust studies, especially longitudinal research that can support evidence-based prevention interventions and policy change. Future studies should focus on increasing their geographical span and sample sizes, ensure the use of standard definitions of alcohol use and AUDs which are consistent with global literature, and acknowledge and examine contextual variations in types of alcoholic beverages and type and size of vessels from which alcohol is poured or consumed in. Introducing such measures will enhance the robustness, validity and generalisability of the findings; and allow for better comparisons over time and geography. This would require greater support from the Government through ensuring availability of in-country research funding, prioritisation of the issue and utilisation of the evidence generated to inform its policy on alcohol.

Our review is limited by our inclusion criterion related to language. However, this might not be a major limitation considering that peer-reviewed journals in India are only in English as far as we are aware, and researchers generally disseminate their outputs in English language journals. Our review's major strength lies in its originality (the first such review to comprehensively map the landscape of substance use among adolescents in India), use of robust processes (e.g. double screening) and examination of grey literature to identify any relevant evidence.

To conclude, the evidence base for alcohol use amongst adolescents in India needs further and deeper exploration, but in the meanwhile, the available evidence allows us to get a preliminary understanding of the issue and to make a case for policy action to tackle alcohol consumption in this age group.

## Supplementary material

For supplementary material accompanying this paper visit https://doi.org/10.1017/gmh.2021.48. click here to view supplementary material

## Appendix A: Search strategy

1. abuse.tw
2. abuse/
3. misuse.tw
4. misuse/
5. use.tw
6. use/
7. disorders.tw
8. disorders/
9. withdraw*.tw
10. withdraw*/
11. withdrawal syndrome.tw
12. withdrawal syndrome/
13. screening.tw
14. screening/
15. overdose.tw
16. overdose/
17. megadose.tw
18. megadose/
19. dependen*.tw
20. dependen*/
21. intoxication.tw
22. intoxication/
23. harm*.tw
24. harm*/
25. hazard*.tw
26. hazard*/
27. behavior.tw
28. behavior/
29. Addict*.tw
30. Addict*/
31. alcoholi*.tw
32. alcoholi*/
33. delirium.tw
34. delirium/
35. binge drink*.tw
36. binge drink*/
37. consumption.tw
38. consumption/
39. drink*.tw
40. drink*/
41. sniff*.tw
42. sniff*/
43. snort*.tw
44. snort*/
45. cessation.tw
46. cessation/
47. smok*.tw
48. smok*/
49. inject*.tw
50. inject*/
51. OR (1–50)
52. Drug.tw
53. Drug/
54. Substance.tw
55. Substance/
56. Alcohol.tw
57. Alcohol/
58. ‘purple drank’.tw
59. ‘purple drank’/
60. 1plsd.tw
61. lplsd/
62. unclassified drug.tw
63. unclassified drug/
64. 2cb.tw
65. 2cb/
66. chlorobenzoic acid derivative.tw
67. chlorobenzoic acid derivative/
68. 4fa.tw
69. 4fa/
70. Ecstasy.tw
71. Ecstasy/
72. methadone.tw
73. methadone/
74. morphine.tw
75. morphine/
76. buprenorphine.tw
77. buprenorphine/
78. diamorphine.tw
79. diamorphine/
80. amphetamine.tw
81. amphetamine/
82. amphetamine derivative.tw
83. amphetamine derivative/
84. AUD.tw
85. AUD/
86. bidi.tw
87. bidi/
88. tobacco.tw
89. tobacco/
90. cigarette.tw
91. cigarette/
92. electronic cigarette.tw
93. electronic cigarette/
94. e-cig.tw
95. e-cig/
96. beedi.tw
97. beedi/
98. benzodiazepine derivative.tw
99. benzodiazepine derivative/
100. benzodiazepine.tw
101. benzodiazepine/
102. bhang.tw
103. bhang/
104. Hashish.tw
105. Hashish/
106. cannabi*.tw
107. cannabi*/
108. ‘brown sugar’.tw
109. ‘brown sugar’/
110. medical cannabi*.tw
111. medical cannabi*/
112. tetrahydrocannabinol.tw
113. tetrahydrocannabinol/
114. hash.tw
115. hash/
116. charas.tw
117. charas/
118. cocaine.tw
119. cocaine/
120. cocaine derivative.tw
121. cocaine derivative/
122. smack.tw
123. smack/
124. crack.tw
125. crack/
126. syrup.tw
127. syrup/
128. chlorpheniramine.tw
129. chlorpheniramine/
130. ‘cough syrup’.tw
131. ‘cough syrup’/
132. codeine.tw
133. codeine/
134. dexamphetamine.tw
135. dexamphetamine/
136. dextromethorphan.tw
137. dextromethorphan/
138. 3,4 methylenedioxyamphetamine.tw
139. 3,4 methylenedioxyamphetamine/
140. psychedelic agent.tw
141. psychedelic agent/
142. ganja.tw
143. ganja/
144. 4 aminobutyric acid.tw
145. 4 aminobutyric acid/
146. 4 hydroxybutyric acid.tw
147. 4 hydroxybutyric acid/
148. GHB.tw
149. GHB/
150. Ketamine.tw
151. Ketamine/
152. glue.tw
153. glue/
154. heroin.tw
155. heroin/
156. nicotine.tw
157. nicotine/
158. diamorphine.tw
159. diamorphine/
160. inhalant.tw
161. inhalant/
162. kava extract.tw
163. kava extract/
164. kava.tw
165. kava/
166. smokeless tobacco.tw
167. smokeless tobacco/
168. khaini.tw
169. khaini/
170. laughing gas.tw
171. laughing gas/
172. nitrous oxide.tw
173. nitrous oxide/
174. LSD.tw
175. LSD/
176. Lysergic acid diethylamide.tw
177. Lysergic acid diethylamide/
178. Acid.tw
179. Acid/
180. Lucy.tw
181. Lucy/
182. magic mushroom.tw
183. magic mushroom/
184. hallucinogenic fungus.tw
185. hallucinogenic fungus/
186. mari#uana.tw
187. marj#uana/
188. MDMA.tw
189. MDMA/
190. Midomafetamine.tw
191. Midomafetamine/
192. amphetamine.tw
193. amphetamine/
194. methamphetamine.tw
195. Methamphetamine/
196. Crystal meth.tw
197. Crystal meth/
198. Amobarbital.tw
199. Amobarbital/
200. Methylphenidate.tw
201. Methylphenidate/
202. Modafinil.tw
203. Modafinil/
204. Morphine.tw
205. Morphine/
206. Opiod*.tw
207. Opiod*/
208. Opiate*.tw
209. Opiate*/
210. Opium.tw
211. Opium/
212. ‘paint thinner’.tw
213. ‘paint thinner’/
214. promethazine.tw
215. promethazine/
216. psilocybin#.tw
217. psilocybin#/
218. Quaalude.tw
219. Quaalude/
220. Methaqualone.tw
221. Methaqualone/
222. Salvia divinorum.tw
223. Salvia divinorum/
224. Psychotropic agent.tw
225. Psychotropic agent/
226. Snuff.tw
227. Snuff/
228. Chewing tobacco.tw
229. Chewing tobacco/
230. Tramadol.tw
231. Tramadol/
232. Viagra.tw
233. Viagra/
234. Sildenafil.tw
235. Sildenafil/
236. Z-class.tw
237. Z-class/
238. Zdrug.tw
239. Zdrug/
240. Eszopiclone.tw
241. Eszopiclone/
242. Zaleplon.tw
243. Zaleplon/
244. Zoipidem.tw
245. Zoipidem/
246. Zopiclone.tw
247. Zopiclone/
248. Hypnotic agent.tw
249. Hypnotic agent/
250. Prescription durg.tw
251. Prescription drug/
252. Prescription medicine.tw
253. Prescription medicine/
254. Prescription medication.tw
255. Prescription medication/
256. OR (52-255)
257. adolescen*.tw
258. adolescen*/
259. child*.tw
260. child*/
261. youth*.tw
262. youth*/
263. student*.tw
264. student*/
265. girl*.tw
266. girl*/
267. teen*.tw
268. teen*/
269. boy*.tw
270. boy*/
271. young adult*.tw
272. young adult*/
273. young*.tw
274. young*/
275. OR (257-274)
276. india.tw
277. India/
278. ‘Indian union’.tw
279. ‘Indian union’/
280. Andaman and Nicobar Island*.tw
281. Andaman and Nicobar Island/
282. Andhra Pradesh.tw
283. Andhra Pradesh/
284. Arunachal Pradesh.tw
285. Arunachal Pradesh/
286. Assam.tw
287. Assam/
288. Bihar.tw
289. Bihar/
290. Dadra and Nagar Haveli.tw
291. Dadra and Nagar Haveli/
292. Chhattisgarh.tw
293. Chhattisgarh/
294. Daman and Diu.tw
295. Daman and Diu/
296. National Capital Territory of New Delhi.tw
297. National Capital Territory of New Delhi/
298. Delhi.tw
299. Delhi/
300. Goa.tw
301. Goa/
302. Gujarat.tw
303. Gujarat/
304. Haryana.tw
305. Haryana/
306. Himachal Pradesh.tw
307. Himachal Pradesh/
308. Jammu and Kashmir.tw
309. Janmu and Kashmir/
310. Janmu.tw
311. Janmu/
312. Kashmir.tw
313. Kashmir/
314. Jharkhand.tw
315. Jharkhand/
316. Karnataka.tw
317. Karnataka/
318. Mysore.tw
319. Mysore/
320. Kerala.tw
321. Kerala/
322. Travancore-Cochin.tw
323. Travancore-Cochin/
324. Madhya Pradesh.tw
325. Madhya Pradesh/
326. Madhya Bharat.tw
327. Madhya Bharat/
328. Maharashtra.tw
329. Maharashtra/
330. Manipur.tw
331. Manipur/
332. Meghalaya.tw
333. Meghalaya/
334. Mizoram.tw
335. Mizoram/
336. Nagaland.tw
337. Nagaland/
338. Odisha.tw
339. Odisha/
340. Orissa.tw
341. Orissa/
342. Punjab.tw
343. Punjab/
344. Chandigarh.tw
345. Chandigarh/
346. Rajasthan.tw
347. Rajasthan/
348. Sikkim.tw
349. Sikkim/
350. Tamil Nadu.tw
351. Tamil Nadu/
352. Madras State.tw
353. Madras State/
354. Telangana.tw
355. Telangana/
356. Tripura.tw
357. Tripura/
358. Uttarakhand.tw
359. Uttarakhand/
360. Uttaranchal.tw
361. Uttaranchal/
362. Uttar Pradesh.tw
363. Uttar Pradesh/
364. United Provinces.tw
365. United Provinces/
366. West Bengal.tw
367. West Bengal/
368. Mizoram.tw
369. Mizoram/
370. Nagaland.tw
371. Nagaland/
372. Lakshadweep.tw
373. Lakshadweep/
374. P#d#cherry.tw
375. P#d#cherry/
376. OR (276–375)
377. 51 AND 256 AND

## Appendix B: Quality of reporting of peer-reviewed studies included in the review (excluding the report)

**Table:**

Author, Year	Title/abstract	Background/rationale	Objectives	Study design	Setting	Participants	Variables	Data sources/measurement	Bias	Study size	Quantitative variables	Statistical methods	Participants	Descriptive data	Outcome data	Main results	Other analyses	Key results	Limitations	Interpretation	Generalisability	Funding
Ahmad *et al*. (2009)	Y	Y	Y	Y	Y	Y	Y	Y	N	Y	Y	Y	Y	Y	Y	Y	N	Y	Y	Y	Y	N
Anandi *et al*. (2018)	Y	Y	Y	Y	Y	Y	Y	Y	N	Y	Y	Y	Y	Y	Y	Y	N	N	Y	Y	Y	N
Armstrong *et al*. (2013)	Y	Y	Y	Y	Y	Y	Y	Y	Y	Y	Y	Y	Y	Y	Y	Y	Y	Y	Y	Y	N	Y
Arya *et al*. (2016)	Y	Y	N	Y	Y	Y	Y	Y	Y	Y	Y	Y	Y	Y	Y	Y	Y	Y	Y	Y	Y	N
Bahl and Kumari (2017)	Y	Y	Y	Y	Y	Y	N	N	N	Y	N	N	Y	Y	Y	Y	Y	Y	N	Y	N	N
Bardhan *et al*. (2015)	Y	N	Y	Y	Y	Y	Y	Y	N	Y	Y	N	Y	Y	Y	Y	Y	Y	N	Y	N	Y
Bashir *et al*. (2015)	Y	Y	Y	Y	Y	Y	N	N	N	Y	Y	N	Y	Y	Y	Y	Y	Y	N	Y	N	N
Bhad *et al*. (2017)	Y	Y	Y	Y	Y	Y	N	N	N	Y	Y	N	Y	Y	Y	Y	Y	Y	Y	Y	Y	N
Deb *et al*. (2013)	N	Y	Y	Y	Y	Y	Y	Y	N	Y	N	N	Y	Y	Y	Y	Y	Y	N	N	N	N
Gaidhane *et al*. (2008)	Y	Y	Y	Y	Y	Y	Y	Y	N	Y	Y	Y	Y	Y	Y	Y	Y	Y	Y	Y	Y	N
Garg *et al*. (2009)	N	Y	Y	N	Y	Y	Y	Y	Y	Y	Y	Y	Y	Y	Y	Y	N	Y	Y	Y	Y	N
Gupta *et al*. (2018)	N	Y	Y	N	Y	Y	Y	Y	Y	Y	Y	Y	Y	Y	Y	Y	Y	Y	Y	Y	Y	Y
Gupta *et al*. (1987)	N	N	Y	Y	N	Y	Y	Y	N	Y	Y	N	Y	Y	Y	Y	N	Y	N	N	N	N
Haorongbam *et al*. (2018)	Y	Y	Y	Y	Y	Y	Y	Y	Y	Y	Y	Y	Y	Y	Y	Y	Y	Y	Y	Y	N	N
Jain *et al*. (2012)	N	Y	Y	Y	Y	Y	Y	Y	Y	Y	Y	Y	Y	Y	Y	Y	Y	N	N	N	N	N
Jaisoorya *et al*. (2016)	N	Y	Y	Y	Y	Y	Y	Y	Y	Y	Y	Y	Y	Y	Y	Y	Y	Y	Y	Y	Y	Y
Jayakrishnan *et al*. (2011)	Y	Y	Y	Y	Y	Y	Y	Y	N	Y	Y	Y	Y	Y	Y	Y	Y	Y	Y	Y	Y	N
Jayakrishnan *et al*. (2016)	N	Y	N	N	Y	Y	Y	Y	Y	Y	Y	Y	Y	Y	Y	Y	Y	Y	Y	Y	Y	N
Kalpana and Kavya (2012)	Y	N	Y	Y	Y	Y	N	Y	N	Y	Y	N	Y	Y	Y	Y	Y	Y	Y	Y	Y	N
Katyal *et al*. (2014)	N	Y	N	Y	Y	Y	Y	Y	Y	Y	Y	N	Y	Y	Y	Y	Y	Y	N	Y	N	Y
Kokiwar and Jogdand (2011)	N	N	Y	Y	Y	Y	N	N	N	Y	N	Y	Y	Y	Y	Y	Y	Y	N	Y	N	N
Kotwal *et al*. (2005)	N	Y	Y	Y	Y	Y	Y	Y	Y	Y	Y	Y	Y	Y	Y	Y	Y	Y	N	Y	Y	N
Kumar *et al*. (2016)	Y	Y	Y	Y	Y	Y	Y	Y	N	Y	N	Y	Y	Y	Y	Y	N	Y	Y	Y	N	Y
Kundapur and Kodyalamoole (2016)	Y	Y	Y	Y	Y	Y	Y	Y	N	Y	Y	N	Y	N	Y	Y	Y	Y	N	Y	N	Y
Lal and Singh (1979)	N	Y	Y	Y	Y	Y	Y	Y	N	Y	Y	N	Y	Y	Y	Y	Y	Y	N	Y	N	N
Mahanta *et al*. (2016)	Y	Y	Y	Y	Y	Y	Y	Y	N	Y	Y	Y	Y	N	Y	Y	Y	Y	N	Y	N	Y
Mandal *et al*. (2019)	Y	Y	N	Y	Y	Y	N	Y	N	Y	N	Y	Y	Y	Y	Y	Y	Y	Y	Y	Y	N
Medhi *et al*. (2006)	Y	Y	Y	Y	Y	Y	Y	Y	N	Y	Y	Y	Y	Y	Y	Y	Y	Y	Y	Y	N	Y
Meshram *et al*. (2015)	Y	Y	Y	Y	Y	Y	N	N	Y	Y	Y	Y	Y	Y	Y	Y	Y	Y	Y	Y	N	Y
Mohan *et al*. (1981)	N	N	N	Y	Y	Y	N	Y	N	Y	Y	Y	Y	N	Y	Y	Y	Y	N	Y	N	N
Mohan *et al*. (1978b)	Y	N	Y	Y	Y	Y	Y	Y	N	Y	Y	N	N	N	Y	Y	Y	Y	Y	Y	Y	N
Mohan *et al*. (1978a)	Y	N	Y	Y	Y	N	Y	N	N	Y	Y	N	Y	Y	Y	Y	Y	Y	N	Y	N	Y
Mohan and Arora (1976)	N	N	Y	Y	Y	Y	Y	N	N	Y	Y	N	Y	N	Y	Y	Y	Y	Y	Y	Y	N
Mohanan *et al*. (2014)	N	Y	Y	Y	Y	Y	Y	Y	Y	Y	Y	Y	Y	Y	Y	Y	Y	Y	Y	Y	Y	N
Mohanty *et al*. (2013)	Y	Y	Y	Y	N	Y	N	Y	Y	Y	Y	Y	Y	Y	Y	Y	N	Y	N	Y	Y	Y
Nadkarni *et al*. (2015)	Y	Y	Y	Y	Y	Y	Y	Y	Y	Y	Y	Y	Y	Y	Y	Y	Y	Y	Y	Y	Y	Y
Nagendra and Koppad (2017)	Y	Y	Y	Y	Y	Y	N	Y	N	Y	Y	N	Y	Y	Y	Y	Y	Y	N	Y	N	Y
Naik *et al*. (2011)	Y	N	N	Y	Y	Y	N	N	N	Y	N	N	Y	Y	Y	Y	Y	Y	N	Y	N	N
Ningombam *et al*. (2011)	Y	Y	Y	Y	Y	Y	Y	Y	N	Y	Y	Y	Y	Y	Y	Y	Y	Y	Y	Y	N	N
Nuken *et al*. (2013)	N	Y	N	Y	Y	Y	Y	Y	Y	Y	Y	Y	Y	Y	Y	Y	Y	Y	Y	Y	Y	Y
Olumide *et al*. (2014)	Y	Y	Y	Y	Y	Y	Y	Y	Y	Y	Y	Y	Y	Y	Y	Y	Y	Y	Y	Y	Y	Y
Pillai *et al*. (2009)	N	Y	Y	Y	Y	Y	Y	Y	N	Y	Y	Y	Y	Y	Y	Y	Y	Y	Y	Y	Y	Y
Pillai *et al*. (2008)	N	Y	Y	Y	Y	Y	Y	Y	Y	Y	Y	Y	Y	Y	Y	Y	Y	N	Y	Y	Y	Y
Rani and Sathiyaskaran (2013)	N	Y	Y	Y	Y	Y	Y	Y	Y	Y	N	N	Y	Y	Y	Y	Y	Y	Y	Y	N	Y
Rathore *et al*. (2015)	N	Y	Y	Y	Y	Y	N	Y	N	Y	N	Y	Y	Y	Y	Y	Y	Y	Y	Y	Y	N
Sandhya *et al*. (2013)	N	Y	N	N	N	N	Y	Y	Y	Y	Y	Y	N	Y	Y	Y	Y	Y	Y	Y	Y	Y
Sarangi *et al*. (2008)	N	N	Y	Y	Y	Y	N	N	N	Y	N	N	Y	Y	Y	Y	Y	Y	N	N	N	Y
Saxena *et al*. (2010)	Y	Y	N	Y	Y	Y	Y	Y	N	Y	N	Y	Y	Y	Y	Y	Y	Y	N	Y	N	N
Sharma *et al*. (2016)	Y	Y	Y	Y	Y	Y	Y	Y	N	Y	Y	N	Y	Y	Y	Y	Y	Y	N	Y	N	Y
Sharma *et al*. (2015)	Y	Y	Y	Y	Y	Y	N	N	N	Y	N	N	Y	Y	Y	Y	Y	Y	N	Y	N	N
Shrivastava and Bobhate (2010)	Y	Y	Y	Y	Y	Y	Y	Y	N	Y	Y	N	Y	Y	Y	Y	Y	Y	N	Y	N	N
Singh *et al*. (2010)	N	Y	Y	N	Y	Y	Y	Y	Y	Y	Y	Y	Y	N	Y	Y	Y	Y	Y	Y	Y	N
Singh and Preet (1981)	N	Y	Y	Y	Y	Y	N	N	N	Y	N	N	Y	Y	Y	Y	Y	Y	N	Y	N	N
Sukhwal and Suman (2013)	Y	Y	Y	Y	Y	Y	Y	Y	N	Y	Y	Y	Y	Y	Y	Y	Y	Y	N	Y	N	Y
Tsering *et al*. (2010)	Y	N	Y	Y	Y	Y	Y	Y	N	Y	Y	Y	Y	Y	Y	Y	Y	Y	N	Y	N	Y
